# Newly arrived Asian-born gay men in Australia: exploring men’s HIV knowledge, attitudes, prevention strategies and facilitators toward safer sexual practices

**DOI:** 10.1186/s12879-022-07174-z

**Published:** 2022-03-03

**Authors:** Tiffany R. Phillips, Nicholas Medland, Eric P. F. Chow, Kate Maddaford, Rebecca Wigan, Christopher K. Fairley, Jade E. Bilardi, Jason J. Ong

**Affiliations:** 1 Melbourne Sexual Health Centre, Alfred Health, Carlton, VIC 3053 Australia; 2 Central Clinical School, Monash University, Melbourne, VIC Australia; 3 The Kirby Institute, University of New South Wales, Sydney, Australia; 4 Centre for Epidemiology and Biostatistics, Melbourne School of Population and Global Health, The University of Melbourne, Melbourne, Australia; 5 Department of General Practice, University of Melbourne, VIC, Australia

**Keywords:** HIV prevention, Homosexuality, PrEP, Asian, Immigrant, HIV-related stigma

## Abstract

**Background:**

Asian-born gay, bisexual and other men who have sex with men (gbMSM) newly arrived in Australia are more than four times as likely than their Australian-born counterparts to be diagnosed with incident HIV. Our aim was to explore experiences of Asian-born gbMSM newly arrived in Australia and attending a sexual health centre with regards to their knowledge of and preference for HIV prevention strategies.

**Results:**

Twenty-four gbMSM aged 20–30 years attending Melbourne Sexual Health Centre who were born in Asia and arrived in Australia in the preceding four years, participated in semi-structured face-to-face interviews from 8th May 2019 and 23rd December 2019. Men were excluded if they were living with HIV. Interviews were recorded, transcribed verbatim and analysed thematically. Men reported little knowledge of HIV prevention strategies outside of condom use prior to coming to Australia. Although participants reported basic knowledge of HIV transmission and treatment, exposure to sexual identity and HIV-related stigma in their countries of birth meant they imagined a HIV diagnosis would be devastating. Most relied on condoms to stay HIV negative however their consistency of use varied. Seven men were on pre-exposure prophylaxis (PrEP); all but one started PrEP after coming to Australia. Many indicated interest in PrEP but described it as too expensive given they do not have access to government-subsidized healthcare. Sexual health counselling and connections with LGBTQI community groups appeared to facilitate PrEP and consistent condom use.

**Conclusions:**

Asian-born gbMSM newly-arrived to Australia may have limited knowledge of HIV prevention strategies aside from condom use. Increased connections with sexual health services and LGBTQI communities may facilitate more effective HIV prevention strategies.

**Supplementary Information:**

The online version contains supplementary material available at 10.1186/s12879-022-07174-z.

## Introduction

HIV diagnosis rates for Australian-born gay, bisexual and other men who have sex with men (gbMSM) are declining since widespread implementation of pre-exposure prophylaxis (PrEP) in 2016 [1]. However, among Asian-born gbMSM living in Australia, HIV diagnosis rates have more than doubled from 2008 to 2017, with men in this group making up 23% of new diagnoses in Australia in 2017 (up from 9% in 2008) [2]. Differential access to government-subsidized healthcare (Medicare)–which greatly reduces the cost of PrEP–may contribute to the increase in the proportion of Asian-born gbMSM diagnosed with HIV [3]. A study of Asian-born gbMSM newly-arrived (within four years) diagnosed with HIV between 2014 and 2017 in Australia found that most (88%) did not have access to Medicare [4]. Additionally, a study of HIV testing among gbMSM in Melbourne in 2017 showed newly-arrived Asian-born gbMSM were less likely to take PrEP than Australian-born gbMSM (6.8% vs 11.1%) [5]. This study also suggested however, that the difference in the proportion of PrEP use between Asian-born and Australian-born gbMSM was unlikely great enough to account for the four-fold difference in HIV diagnoses between these two groups, and that other factors may contribute to this disparity, such as restricted access to HIV testing and treatment [5].

Despite the rising rates of HIV among newly-arrived Asian born gbMSM in Australia, this group has reported fewer numbers of casual sex partners and less condomless anal sex with casual partners than their Australian counterparts [4]. It is possible that the lower reported risk factors are in part due to increased internalised sexual identity and HIV-related stigma, as studies indicate both are prevalent in various countries in Asia [6–14]. Stigmatising attitudes toward HIV are associated with increased sexual risk behaviours [9, 15].

The primary aim of this study was to examine the experiences of Asian-born gbMSM newly arrived in Australia and recruited from a sexual health clinic, specifically in terms of their knowledge of and attitudes toward HIV, HIV prevention strategies and facilitators of safer sexual practices.

## Materials and methods

Data were reported in accordance with the consolidated criteria for reporting qualitative research (COREQ) checklist. [16].

### Theoretical framework

A social constructivist approach informed the framework of this study. From a social constructivist viewpoint, an individual’s experience of reality and the meaning they give to phenomena (values, beliefs, experiences) are influenced by societal and cultural norms operating at the time. [17] From a social constructivist perspective, their ‘constructions’ of the world, or ways of knowing and being in the world, are produced through the interactions with other people, places, systems and beliefs that exist at any one point in time. In more conservative Asian countries, considerable stigma still surrounds both same-sex attraction and HIV [6, 8, 10, 11]. From a social constructivist perspective, Asian born MSM’s knowledge, views and practices around HIV are likely to be heavily influenced by these cultural and religious beliefs, attitudes and norms pertaining to sexual identity and HIV in their country of origin [18].

### Method, research team and reflexivity

Once off, semi-structured interviews were undertaken for men to share their lived experience of being a gbMSM. All interviews were conducted by TP, a female research fellow in sexual health with experience conducting qualitative interviews. Participants had no prior relationship with TP and were informed that the purpose of the study was to understand HIV prevention strategies preferred and utilised among newly-arrived Asian-born gbMSM in Australia due to the increasing rates of HIV diagnoses in this population. An interview schedule was developed by the research team which was informed through existing literature and clinical and academic expertise in the area. JB assisted TP with data analysis. JB is a senior research fellow who has extensive experience in conducting qualitative sexual and reproductive health research.

### Recruitment

Men were purposively sampled to explore possible differences in experiences. The sampling framework is detailed in Table 1. Participants were recruited from the Melbourne Sexual Health Centre (MSHC) between 8th May 2019 and 23rd December 2019. MSHC is the largest public sexual health clinic in Victoria, Australia. Clients attending MSHC were invited to complete a computer-assisted self-interview (CASI), which collects information on their demographics and sexual practices. Men indicating that they were born overseas and had had sex with a man in the preceding 12 months were shown an automatically computer-generated invitation to take part in the study. Men interested in participating left their preferred contact details (SMS or email) and a research nurse (KM or RW) contacted them to ensure eligibility and schedule an interview. Men were then asked for their email and were emailed the plain language statement. Informed written consent was obtained in person when men returned to the recruiting clinic for the interview.

**Table 1:** Sampling framework, eligibility criteria and interview schedule topics

Sampling framework
Gay, bisexual and other men who have sex with men (gbMSM)
Recently arrived in Australia (less than five years ago) from an Asian country, defined as: Bangladesh, Bhutan, Brunei, Cambodia, China, East Timor, Hong Kong, India, Indonesia, Japan, North Korea, South Korea, Laos, Macau, Malaysia, Maldives, Mongolia, Myanmar, Nepal, Pakistan, Philippines, Singapore, Sri Lanka, Taiwan, Thailand or Vietnam
Clients of Melbourne Sexual Health Centre
HIV negative
Range of ages
*Revised during data collection to include:*
More men from countries outside of China
Eligibility Criteria
Male
Aged 18 years or older
Good understanding of English
Interview schedule topics
Experience of being gbMSM in country of origin^a^
Experience of being gbMSM in Australia*
HIV and STI knowledge
Preferred HIV prevention strategies
*Revised during data collection to include additional questions*
How HIV is viewed in participant’s country of origin*
Changes in sexual behaviour or prevention strategy since coming to Australia
Participant’s perceptions of HIV risk

^a^Reported in a separate manuscript [18]

### Data collection

Interviews were conducted face-to-face, in English, in a private room at MSHC. All interviews were digitally recorded. Immediately following the interviews TP recorded notes detailing contextual information about the interview and participant including non-verbal cues and other relevant details pertaining to the interview. Participants received an AUD$30 (US$20) voucher following completion of the interview to compensate them for their time. Interviews were transcribed verbatim for thematic analysis. Participants were informed that they could request a copy of the study transcript for member checking, however all participants declined.

### Data analysis

The study team (TP, KM, JB, JO and NM) met regularly to review interview transcripts, discuss the data emerging from the interviews and to revise the interview schedule. After two interviews the research team met to discuss emerging themes and revised the interview schedule to include additional questions on HIV in their countries of origin and changes in sexual behaviour after arriving in Australia. After seven interviews were conducted the team further refined the interview schedule to include questions about participants’ perceptions of HIV risk (Table 1). Regular meetings to discuss the data and developing themes and sub-themes continued until 24 interviews were complete. At this time, TP and JB met to again review the data and agreed that no new themes were emerging and that data saturation had been met.

The data were thematically analysed whereby the transcripts were imported into QSR International’s NVivo 12 software for data management. They were read and coded, grouped into potential themes and subthemes by TP, before being re-read and further refined. A subset of transcripts was read and coded by JB to confirm coding and analysis. Consensus was met between JB and TP on coding and themes and no differences in interpretation were identified between the researchers.

Descriptive analysis of demographic information was performed using Stata (version 14.0, College Station, TX: StataCorp LP). All methods were performed in accordance with the relevant guidelines and regulations.

## Results

Overall, 117 men registered their interest in participating in the study, 97 of whom met the eligibility criteria. Of those eligible, 38 scheduled an interview and of these men 24 completed an interview before data saturation was met. The average length of an interview was 42 min (range 25–57). Participant demographics are reported in Table 2.

**Table 2:** Participant Demographics (*N* = 24)

	***n* or median [range]**
Age (years)	27 [20–34]
Sexual identity^a^	
Gay	21
Bisexual	1
In between gay and bisexual	1
Queer	1
Country of origin	
China	5
Indonesia	3
Malaysia	3
Laos	2
Philippines	2
Singapore	2
Taiwan	2
India	1
Pakistan	1
Sri Lanka	1
Thailand	1
Vietnam	1
Length of time in Australia	
< 12 months	5
1 year	6
2 years	3
3 years	5
4 years	5
Occupation	
Postgraduate student	10
Undergraduate Student	5
Diploma course student	3
Retail	2
Hospitality	3
Unemployed	1

^a^Participant sexual identity was self-reported and listed here verbatim

Three major themes and seven subthemes emerged from the study (Table 3).

**Table 3:** Themes and subthemes

1. Coming to Australia	2. HIV prevention strategies	3. Facilitators and motivators for change in sexual practice
1a. Knowledge about HIV and sexual health 1b Terror of getting HIV 1c. Sexual freedom	2a. Condom use 2b. PrEP	3a. Friend support 3b. The role of counselling and LGBTQI-specific education

## Coming to Australia

### 1a. Knowledge about HIV and sexual health

At the time of the interview all participants reported a basic knowledge about HIV and how it is transmitted. Men commonly spoke about HIV being a virus which impacted the immune system and was spread through bodily fluids like semen and blood.

Yeah HIV is transmitted from body liquid like semen and saliva as well as some other body liquids like blood and the liquid that you emit from some other sexual organs, like prostate or something.

—Participant 13, China, 1.5 years in Australia

Most men’s sexual health knowledge was influenced by the conservative cultures in their countries of origin where social taboos often precluded the discussion of sex. Where sexual education was provided, it was primarily discussed and viewed only in the context of heterosexual sex within marriage. As a consequence, men spoke about seeking same-sex sexual health information from the internet or through their friends.

We don't have this education over there for gay men. So I have to find out through the internet, so *[I]* research by myself and ask about my friends really as well. So, yes, mostly we find it by ourselves. We have a sex-ed but mostly man to girl so we don't kind of get the exposure on men with men.

–Participant 20, Malaysia, 3 years in Australia

### 1b. Terror of getting HIV

Due to the heavily stigmatised view of HIV most men were exposed to in their countries of birth, men commonly reported they were terrified or extremely anxious about acquiring HIV. Their fears centred on the perceived impact it would have on their relationships, their lifespan, quality of life, the potential for onward transmission and concerns about living as a person with HIV in their country of birth (Additional file 1: Table S1). Even participants who knew rationally they could live a normal life with HIV, were unable to separate entirely from the fear of getting HIV, the stigma surrounding it and how they perceived it would impact on their life and, specifically, their romantic relationships.

*…in terms of managing HIV positive, I feel I would be quite alright, but in terms of dealing with the external things around me, so things like colleagues, workplace, yeah, people, family, I think that would be the challenge*

—Participant 11, Malaysia, 4 years in Australia

### 1c. Sexual freedom in Australia

Despite men’s acute fear of contracting HIV, some reported that their limited knowledge of HIV prevention coupled with their desire to explore their sexuality in Australia meant that they engaged in unsafe sexual practices after arriving in Australia. Men often reported having more sex partners after they arrived, with a handful of men reporting they had their first same-sex sexual encounter in Australia. The desire to explore their sexuality freely when they arrived in Australia, meant some men had sex in their early days of living in Australia that they later realised was putting them at risk of HIV and other STIs.

*Because I didn’t think about anything, I just have sex. You know, to use condoms or whatever, I didn’t think about anything. I didn’t even check whether they are negative or positive, or whether they are getting PrEP or PEP or whatever. I didn't check that…*

—Participant 15, Sri Lanka, 2 years in Australia

Further example quotes are shown in Table 4.

**Table 4:** Example quotes on participants knowledge levels of HIV and sexual health and experiences of sexual freedom upon arriving in Australia

Theme 1: Coming to Australia	Example quote
1a. Knowledge about HIV and sexual health	*Well, it’s [HIV] a virus, yeah, which can – it’s so difficult to explain. It’s like AIDS, I think. So, if the virus gets into your body, it will destroy your immune system, yeah, that’s what I know from the internet* —Participant 5, Taiwan, 3 years in Australia
1b. Terror of getting HIV	*Because it limits your span of life here and it's like your life's going to be limited and people are not going to treat you the same as before* —Participant 24, Philippines, 2 years in Australia
1c. Sexual freedom	*For the past few years, for the last year, like crazy, like we had a party, so drunk and I went to the sauna… I had sex with five men, I know it’s so risky, oh my gosh… At that time, I … didn’t know PrEP. Lucky the recent one, he’s the white guy, the western guy—he’s my recent sex partner now, he advised me, you should have the PrEP otherwise it’s going to be so risk, getting HIV Aids. I’m lucky I haven’t got anything* —Participant 4, Thailand, 4 years in Australia

## HIV Prevention Strategies

The primary HIV prevention strategies of men were varied and changed over the course of their time in Australia as their sexual health knowledge levels increased and they became more aware of alternatives such as PrEP. Table 5 summarizes men’s HIV prevention strategies at the time of the interview.

**Table 5:** Condom and HIV pre-exposure prophylaxis (PrEP) use among Asian-born men who have sex with men (*N* = 24), Australia (2020)

	Number
Current sexual partners^a^	
Regular partner only	6
Casual partners only	11
Regular and casual partners	7
On PrEP	7
Condoms^b^	
Always uses condoms	4
Frequently uses condoms	7
Infrequently uses condoms	5
Did not engage in anal sex	1
Reasons for non-PrEP use among men who reported always using condoms (n = 4)	
Too expensive	2
Worried about side effects of the medication	1
Worried about getting other STIs so prefers condoms	1
Reasons for non-PrEP use among men who do not always use condoms (n = 12)	
Too expensive	4
Worried about side effects of the medication	2
Did not understand PrEP before the interview	2
Told by a physician that not at risk of HIV; very few sexual partners	1
Partner on PrEP, feels he doesn’t need it	1
Has a monogamous relationship so feels does not need it	1
No specific reason given	1

^a^Current sexual partners at time of the interview

^b^Condom use describes the current level of condom use the participants reported at the time of the interview. These numbers do not reflect change in practice over time in Australia (i.e. those with more or less condom use over time)

### 2a. Condom use

On arriving in Australia, most men relied on condoms to prevent HIV. For many, this was because condoms were the only form of HIV prevention they knew about.

At the time of the interview, there were three distinct groups of men reporting condom use (if they were not on PrEP): those who always used condoms, those for whom condomless sex was the exception to the rule (frequent users), and those who infrequently used condoms (infrequent users). Reasons men reported having sex without protection against HIV varied (see Table 6 with example quotes), but most generally centred around trust; trust in their casual or regular sex partner to be HIV negative, as well as trust in their own intuition when choosing a sex partner.

**Table 6:** Representative quotes describing reasons for not using condoms consistently among those not on PrEP at the time of the interview

Men who reported infrequent condom use in Australia	
*Trusted partners to disclose HIV status*	*Usually I’ll ask the partner first [if they’re fine not using a condom]. Yeah so if they’re fine with it then I guess, yeah, I won’t use… Yeah, I don’t ask [if a person is HIV positive before having sex] … I guess it’s like, kind of, their obligation to tell you that they have it. Because it’s a transmittable disease and it’s quite deadly. And if they know about it they should inform you. Yeah* –Participant 23, Singapore, 8 months in Australia
*Trusted his intuition*	*I would be more worried about STIs… I don’t really feel like there are so many HIV positive people, and I am selective when I am doing it, when I was doing it* —Participant 7, China, 1.5 years in Australia
*Trusted partner to be monogamous*	*The first time [having sex with his regular partner] and the second time I used a condom. But the third time, he don't want to. First time he did not want to, I really don't like the way, I want to use the condom. Lack of protection. He doesn't… he said he was on PrEP and that he is clean and he got tested. I said okay. Maybe yes. What happened to me? Sometimes it was just impulse… I never tried that like without condoms. Maybe I was curious… I told him, if you want to do that this way, you mustn't meet another person* –Participant 17, China, 3 years in Australia
*Believed men in his social circle did not have HIV*	*In Pakistan, I don’t use condoms because the circles I move in are the circles which are the people like me… like, my age group, and they are from nice families and things like that. So there is not HIV or many sexually diseases, transmitted disease in Pakistan. After I moved here, at the start I didn’t use condoms…because it is a mental habit…* —Participant 1, Pakistan, 2.5 years in Australia
*Lacked knowledge of STIs or HIV*	*Because I didn’t think about anything, I just have sex. You know, to use condoms or whatever, I didn’t think about anything. I didn’t even check whether they are negative or positive, or whether they are getting PrEP or PEP or whatever. I didn't check that, I just…Yeah, I didn’t have any knowledge* —Participant 15, Sri Lanka, 2 years in Australia
*Let sex partners decide*	*Because sometimes the person doesn’t want to use it. So, I just follow* —Participant 5, Taiwan, 3 years in Australia

Men who reported frequent condom use in Australia	
*Let sex partners decide*	*Mostly [I] will use a condom but there's a few times that [a partner] didn't want to. A few times—two times. Yes. They didn't want to. But when I do it I don't really quite feel comfortable to it* -Participant 20, Malaysia, 3 years in Australia
*Believed it was partners first time having condomless sex*	*I actually [give] some kind of interview [to] him first like talking, is this your first time, is this your—because I have this instinct that it's his first time [having sex without a condom] I guess so we're just going to try it without condom. So based on what he's saying about anything I think it's [his] first time…So, yes, we're not going to use a condom. But when I know a person is already doing it [casual sex without a condom] for a longer time already so we're going to use condom…. We just kind of talk about if you have been to other casual sex without using a condom before* —Participant 24, Philippines, 2 years in Australia
*Trusted intuition*	*Usually, I talk to him and I get to trust him and also ask him if he’s negative or something. Yes I think it’s not really, yes it could still be risky but sometimes I just follow my sense, yeah* —Participant 13, China, 1.5 years in Australia
*Loved and trusted partner*	*I think I really loved him. I think with this love and affection, you might think, “Okay. You can trust him with anything.”* —Participant 10, Taiwan, 4 years in Australia
*Hooked up more than three times*	*I always use condoms unless I’ve had contact with the guy for more than three times* —Participant 8, Indonesia, 7 months in Australia
*Partner passed PrEP quiz*	*I test, test about PrEP. Yeah, test about price, about the brand of drugs, like I test it. If they pass the test, I ask – yeah [I would consider not using a condom]; I have two experience without condom, but [the casual partner was] on PrEP* —Participant 14, Indonesia, 3 years in Australia

Most men described instances of condomless sex during their time in Australia. However, a small number of men described always using condoms to prevent HIV with no instances of condomless sex; generally saying they felt safer using condoms even when a partner said they were on PrEP. For these men, the risk of acquiring HIV felt too great and they were unwilling to have condomless sex in any circumstances, regardless of partner type (i.e. regular or casual).

### 2b. PrEP

Of the 24 men interviewed, seven were currently taking PrEP; only one of whom initiated PrEP in their country of birth (Singapore). Most men on PrEP also used condoms to some degree, particularly with casual partners as they were still quite anxious about acquiring HIV. Men described PrEP as an extra ‘shield’ or barrier against HIV, noting that in situations where condoms are not desirable, they have a level of protection with PrEP.

*I’m on PrEP and I use condoms as well to prevent myself from getting HIV…I don’t know, I just felt because I was quite sexually active so I just wanted to safeguard myself more against all these diseases. Your life could just go just like that and I don’t want it to be because of HIV.*

—Participant 16, Singapore, 3 months in Australia

Among PrEP users, most were in a regular relationship and had casual partners as well. All but one had agreements with their regular partners about sex outside of the relationship and had some form of negotiated safety, which generally meant using condoms always or frequently with casual partners but not with each other.

*So, basically, the idea is that—so, we’re not going to use condom between us, but whenever we have sex with anyone else, we’re going to use condom, and both of us are on PrEP.*

—Participant 21, Vietnam, 3 years in Australia

The exception to these negotiated safety arrangements was one participant who did not have an agreement to see other people outside of his regular partnership. His regular partner was not on PrEP and did not want the participant to remain on PrEP. They both disclosed they had been “cheating” by having casual sex with other men after being together for one year and his partner felt he could not trust him if he remained on PrEP. The participant was planning to get tested for HIV and stop taking PrEP at the time of the interview.

So I met a guy which was not my partner but at the end I decided to tell him. So I told my partner and he got upset and he told me as well, he says he cheat on me as well. So we both make mistake, so he made mistake as well. Then now, I want to do the blood test [to check for HIV and STIs], I don’t know, he doesn’t feel good that I’m taking PrEP, he doesn’t trust me. So today, I want to do the blood test, check everything all good so now we’ll be back to [only having sex with] each other and I probably [won’t] take PrEP anymore. [I will] just make sure he check his health as well. .. We both honest to each other, we don’t cheat on each other so it won’t happen. *So I have no choice, so I’m going to stop using PrEP and be a good boy.*

—Participant 22, Laos, 1 year in Australia

Another participant (who only had casual partners) was so anxious about getting HIV and STIs that he used PrEP and condoms for anal sex and avoided oral sex altogether.

*I don’t do a lot of oral involvement if it’s casual sex because there are a lot of things which can get transferred with the oral things. Not a lot of people know that. So I prefer not to get involved in the oral thing. If I’m just having casual sex, then I prefer only anal things if there is a condom.*

—Participant 6, India, 5 months in Australia

Six men reported they were interested in PrEP but felt that it was out of reach due to cost (see Table 5). Half were students and the other half were working in retail or hospitality and were unable to access PrEP due to a lack of Medicare access in Australia which would substantially subsidize the costs and make treatment accessible to them.

I do have other expenses that I need to prioritise other than PrEP. One of the options is actually just to buy condoms, you know, it’s cheaper than PrEP monthly. I actually tested already. I’m eligible. My GP already gave me the prescription. I just had to order it. But I stopped myself because it’s just so expensive. *I cannot access it… I could understand why people easily use PrEP here because of Medicare and stuff but for us, it’s expensive. We cannot reach that even though we do really want to.*

—Participant 8, Indonesia, 7 months in Australia

I want to get in PrEP in Australia, but I’m not a resident, it’s expensive for me.

—Participant 14, Indonesia, 3 years in Australia

For some men, the prohibitive cost of PrEP meant that they instead relied on other, less preferred HIV prevention strategies, such as reducing partner number and using condoms.

We [my partner and I] talked about it, especially before I actually asked for a prescription, like I told him, “I’m going to take PrEP instead” before I knew how expensive it was. I thought I could tweak it a bit with a concession price or it turns out to be my insurance not covering PrEP. So we talked about it and it was like, “Why do you want to take PrEP?” “Just because I want to stay negative and once we meet again, we are clean, you know, it’s secure for us to have sex.” So nowadays it’s just like – personally … I choose to have less sex and then less random hook-ups…have sex with someone that I really know already and use condoms. *So that’s how I feel, to make myself stay clean from both STIs and HIV.*

—Participant 8, Indonesia, 7 months in Australia

Others were hesitant to consider PrEP due to the perceived potential side effects. Participants worried about specific effects on their liver, kidneys, and digestive system, while others were not sure exactly what side effects PrEP would have but worried any regimen that involved taking a daily pill would result in unwanted side effects.

But another suggestion from my friend actually when you're taking all those pills actually damaging your liver. *So I'm kind of like should I do this or should I not?*

—Participant 20, Malaysia, 3 years in Australia

PrEP has some side effects so I would avoid using PrEP.

—Participant 10, Taiwan, 4 years in Australia

Several participants described learning about PrEP in Australia and while some felt they knew about the basics of PrEP, they did not feel they knew enough about PrEP to make an informed decision on whether or not they should take it or how it should influence their sex practice.

Maybe I don’t understand PrEP that well. Should I take PrEP? You take PrEP, then we go raw without condoms or not? *That’s the thing that I don’t know.*

—Participant 8, Indonesia, 7 months in Australia

## Facilitators and motivators for PrEP and consistent condom use

Many men described changing HIV prevention strategies to reduce their risk after arriving and during their time in Australia. For some, this change occurred in the form of learning about and taking PrEP for the first time, while for others it resulted in increased or consistent condom use. Changes largely occurred as a result of increased knowledge of HIV risk and prevention strategies which were facilitated by increased social connections and sexual health counselling.

## 3a. Friend support

Most men reported that a friend or sex partner had told them about the recruiting sexual health clinic and that prompted them to get tested for HIV and STIs. For several men, they assumed such a clinic would cost money or would not be covered by their overseas health insurance and thus would otherwise not have come.

I didn't know where and all I know is that when you come to a clinic you have to pay so I didn't really look up into it until my friend told me. *He's American*… He said "you can go to this place. Actually it helps. It's complimentary and it's giving you some information." *That was six months ago as well so one month ago I decided to come because I know I think I should do it to have a check-up.*

—Participant 20, Malaysia, 3 years in Australia

A couple of men had never been tested for HIV before and attending a sexual health clinic was not something they had planned on doing before a friend suggested it.

*The first time I came here was because it was free, but I was kind of reluctant coming here, because I don’t know how people would see me…A friend told me [about the sexual health clinic], and I thought, I haven’t done that, I just really want to check out if this place is good, then I came.*

—Participant 7, China, 1.5 years in Australia

One participant in particular, described how revealing his sexual identity to his first friend was a ‘turning point’ for him since his friend encouraged him to attend the recruiting sexual health clinic for his health and to find his community—a suggestion that led him to a local LGBTQI support group. To him, opening up to a friend about his sexual identity led him to not only learning about sexual health but also to feeling accepted as part of the gay community.

*I didn’t come out until October, it means, like, I come out one year ago, to one of my friends, he just hugged me and told, like, that’s alright, so that’s the day I realised that alright, I shouldn’t be act as a straight person, I should be myself. So, that was the turning point, and that’s why I come to here, I come to MSHC or whatever, because of him. He taught me that you’re normal… Everything done by my friend, he told me to find your community. If I wanted to [give advice to someone in my situation it would be] tell someone, just come out to one of your friend, and you know, like, someone you can [trust]… the first thing, I think, is the knowledge about everything – STIs, but the other thing is, you know, at least one person who knows about yourself is really important.*

—Participant 15, Sri Lanka, 2 years in Australia

While most participants described learning more about sexual health after arriving in Australia, one participant described how his initiation to sexual health was facilitated by the support of a friend in his country of origin. An openly gay friend in China encouraged him to be tested for HIV and accompanied him to have the test since he was very afraid of the experience and had never been tested before. As he described, support from a friend helped him learn about his sexual health and encouraged him to embrace his sexual identity; a step he felt was very important to maintaining sexual health.

…one of my friends, like in the undergraduate, he is very open, he's openly gay. He told me a little bit about that you have to protect yourself. You have to test regularly... I fear about the test or something… The first time I got tested [for HIV] my friend, [accompanied] me. *I feel more comfortable to do that, so yeah…When you first accept you're gay and you know who you are and you [learn] you have higher risk, like to get some infection, you do feel like necessary, oh I have to go to test, I have to use a condom to protection and maybe I have the obligation to tell other gay people and my gay friends to do the same things.*

—Participant 17, China, 3 years in Australia

## 3b. The role of counselling and LGBTQI-specific education

One of the biggest drivers for sexual practice change was an increased awareness and knowledge of PrEP and consistent condom use. Of the men on PrEP at the time of the interview, three had learned about and decided to start PrEP after receiving counselling at the recruiting sexual health clinic and another had learned about it during a consultation with a doctor at the recruiting clinic. Two initiated PrEP after attending sexual health workshops provided by community organisations for people who are LGBTQI (one in Victoria, and another in Singapore—the sole participant who had initiated PrEP in his country of origin). The final participant on PrEP learned about PrEP after attending sexual health education workshops provided by his university in his country of origin, however he felt he could only access PrEP after arriving in Australia and attending the recruiting sexual health clinic as he did not think it was prescribed in his country of origin. Thus, all men on PrEP initiated PrEP after connection to a sexual health service and most had counselling or LGBTQI-specific support. Similarly, those who began using condoms consistently after arriving in Australia credited counselling and LGBTQI organisations for educating them on sexual health. These men described the benefits of sexual health counselling and involvement with LGBTQI services not only in terms of increasing their knowledge of STI and HIV prevention, treatment and support services, but also imparting on them the importance of embracing their sexual identity. For one participant the counselling session made him see how risky his sex practices were given that he never thought to use condoms for anal sex. For him, HIV was not something he ever considered he could get and through counselling he learned about the actual risk and explored what it would mean for him if he were to be infected with HIV.

*I had counselling and that [counsellor told] me all the horrors of HIV and if I have to go back to Pakistan and there will be no, very advanced medicine there, and it is very, very expensive to [get] HIV medicine to get in Pakistan and things like that. I made a conscious decision to go on condom.*

—Participant 1, Pakistan, 2.5 years in Australia (consistent condom user now)

A couple of participants also described benefiting from involvement with LGBTQI community organisations in terms of establishing social connections with people who can relate to them because they are going through similar struggles.

It’s hard for a straight person to relate to a gay person’s feelings because they can’t really put themselves in our shoes. Even though how understanding they might want to be or how much they want to be there for you. It’s not the same as talking to a gay person itself, because the struggles and what they feel is more in line as to what you’re feeling. I feel it’s really nice to meet like gay people that you can really bond and be really close with to just pour your feelings out to. *I’m glad I’ve met them through these events yes.*

—Participant 16, Singapore, 3 months in Australia (on PrEP after involvement in LGBTQI organisation in Singapore)

One participant initially heard about PrEP from the internet but did not imagine it would be right for him. He worried about the side effects of PrEP as well as the routine of needing to have regular check-ups on PrEP. It wasn’t until he had an experience of condomless sex where he forgot to use a condom in the heat of the moment and he attended a workshop run by a community organisation for people who are LGBTQI that he realised he cannot always be in control of using condoms. He made the switch to PrEP to avoid getting caught up in the moment and having condomless sex again in the future.

*Mostly [I heard about PrEP] from the internet…I wasn’t ever actual[ly] think[ing] about that [taking PrEP]. Then, I got to know that more, when I went to one of the workshop at the Victorian AIDS Council…[at that] point I realised there’s no way I’m in control of that [condomless sex in the heat of the moment]. At first, I guess, the idea that, like – oh, there might be side effect, and you know, and also, it [PrEP] might potentially affect your kidney function. Then, also, it’s part of the routine that you basically have to check in, and I have a feeling that I might not be able to keep up with that routine. In a way, I was slightly a little bit discouraged with that, but then, when I had that experience [of condomless sex] I realise I better get on that [PrEP], rather than find myself in that situation again.— Participant 21, Vietnam, 3 years in Australia*

For one participant, involvement with a state-based organisation that supports LGBTQI communities facilitated not only his sexual health knowledge in terms of learning about using PrEP and the risks associated with condomless sex, but his understanding of what it means to be gay and what relationships could look like. Prior to his involvement with this organisation he did not know that he could be gay and also have a family one day or that relationships could be open.

*…after I come out to my friend, he told [me]… I should learn about myself first, that’s what he told [me]. Like, you must go with your own community…So, that advice lead me to go for Thorne Harbour [a local LGBTQI organisation in Melbourne], and I went to three workshops in there…They was amazing, so amazing, because you’re like – it talked about sex, and lifestyles, and relationships, and to be friends, and hang out, and how you’re going to live in the public. It’s like, we are going for bowling, to cinemas sometimes, it’s really – you know that knowledge gives us that…you are a human being, also, and just be yourself…After attending to my workshops, I learnt how to do safe sex, what does unsafe sex mean, what does the PrEP mean, what is the PEP mean, what is the vaccinations. I think that knowledge is really important… Sometimes, we know who we are, but we don’t know who we really are. You know, like, you can make a family, if you want to do open relationship or closed relationship, or whatever. So, I never had that type of idea about these kinds of things.*

—Participant 15, Sri Lanka, 2 years in Australia (Consistent condom user now)

## Discussion

In this study exploring sexual practices and HIV prevention strategies among newly-arrived Asian-born gbMSM recruited from a sexual health centre, we found that most arrived in Australia with only basic knowledge about HIV. Most initially relied on condoms to prevent HIV, with frequency of use varying, and most reported experiences of condomless anal sex in Australia, despite a strong fear of acquiring HIV. Among infrequent condom users, some reported relying on their intuition when deciding to use a condom or not, basing their decision on preconceived and inaccurate notions of HIV and trust in their partner. Major facilitators to changing men’s sexual health practices were sexual health counselling and involvement with LGBTQI-specific community groups. Most PrEP and consistent condom users adopted these practices after involvement with such services. Participants engaging with counselling and LGBTQI organisations increased their awareness of HIV risk, HIV prevention strategies, and the importance of embracing their sexual identity.

Almost all participants were prompted by friends or sex partners to attend the recruiting sexual health clinic for their first time. For some, the clinic was their first introduction to sexual health and testing for HIV and STIs. All but one PrEP user had decided on PrEP after sexual health counselling or attending sexual health workshops provided by organisations that support LGBTQI communities; the other was encouraged by a clinic doctor. Participants who were on PrEP disclosed more negotiated safety practices within their regular partnerships than those who relied on condoms; describing an open dialog about sex and HIV prevention strategies outside and inside the relationship with their regular partner. Negotiated safety is a particular type of sexual agreement within partnerships that typically entails condom use with casual partners outside the relationship. A previous scoping review found that having a sexual agreement with partners (beyond negotiated safety) was associated with engaging in condomless anal sex within the relationship and outside the relationship in several studies [19]. Similarly, Findings from repeated cross-sectional surveys in Australia from 2013 to 2018 found that PrEP use was associated with agreements between partners that allow condomless sex with casual partners, with almost 40% of PrEP users in relationships having such agreements [20]. While participants in our study who were on PrEP described negotiated safety within their partnerships, further research is required to understand whether there is an increase in other types of sexual agreements which allow condomless anal sex in this population. Such research would be important to inform rates of STIs.

This study suggests that central to increasing HIV prevention strategies within this group is reducing their feelings of stigma related to sexual identity and HIV, which may be facilitated by increasing connections with sexual health services and organisations that support LGBTQI communities. Our findings reinforce peer support as an important facilitator to sexual health [21, 22] and indicate that one-on-one counselling and involvement with organisations that support LGBTQI communities can increase knowledge levels and implementation of PrEP and condom use as well as enhance acceptance of sexual identity. Our findings reflect those from a study of 763 young Asian and Pacific Islander men who have sex with men in the United States in 2011, which found that positive dual identity (attitudes toward both their sexual identity and their ethnic identity) was associated with greater social support and HIV testing. [23] Further research on the effectiveness of peer support and involvement with organisations that support LGBTQI communities for increasing sexual health and how these services can be expanded to further reach this population is warranted.

Participants in our study had several misconceptions about their HIV risk upon arrival in Australia, such as believing they could sense when a potential sexual partner has HIV based on their hygiene, social circle, or appearance. Some participants felt they could trust their intuition or partners to keep from getting HIV. These findings reflect previous qualitative work among Chinese GBMSM which found that trust in partners was a barrier to HIV testing and that despite being an important component of healthy relationships, was a point of threat considering rising rates of HIV and low HIV testing rates in this population [24]. Further research on HIV risk perception among newly arrived GBMSM to Australia is warranted to inform sexual health education from clinicians to this population.

There were several limitations that should be noted. Firstly, it is possible participants may not have reported actual risk due to social desirability bias. Secondly, participants were exclusively recruited from a sexual health clinic and were therefore already connected into a sexual health service in some capacity. Our findings may not reflect men who are not getting tested for HIV and other STIs. This limitation highlights the importance of targeted future research into methods of increasing engagement and undertaking larger scale studies on sexual health among newly-arrived Asian-born gbMSM. Similarly, these interviews were conducted in English and therefore do not include experiences of men who do not speak English and may be further marginalised. Our participants were also relatively well-educated, and it is possible those with less education have less sexual health knowledge. However, a high proportion of new HIV diagnoses in Australia are among international students [4], so insights from this population are particularly important. This study provides key insights into how gay and bisexual men who were newly arrived in Australia became connected to sexual health, as well as how their HIV prevention strategies change over the course of their time in Australia as they become connected to sexual health.

## Conclusions

Future studies should focus on ways to reduce internalised sexual health and HIV-related stigma and shame in this population, such as, facilitating peer support groups, offering counselling services for mental and sexual health, and enhancing connections with LGBTQI community groups. It is not known what proportion of men in this population are offered counselling at this or other sexual health clinics in Australia. Given the lower reported risk from this population it may be difficult to identify those men who could potentially benefit from such services, a challenge future research will have to take into consideration.

## Supplementary Information

**Additional file 1: Table S1. **Example quotes of participants’ fear of HIV and the impact a HIV diagnosis would have on their lives.

## Data Availability

Due to the small sample size and the interview transcripts containing sensitive and potentially identifying information, the ethics committee have not approved public release of this type of data. Interested researchers may contact the Alfred Hospital Ethics Committee if they would like access to the data: research@alfred.org.au, quoting project 222/19.
