# Supplementary material for: Newly arrived Asian-born gay men in Australia: exploring men’s HIV knowledge, attitudes, prevention strategies and facilitators toward safer sexual practices
Source: BMC Infect Dis. 2022 Mar 3;22:209. doi: 10.1186/s12879-022-07174-z (PMC8892798; doi:10.1186/s12879-022-07174-z)
Supplement: Supplementary file 1 — Additional file 1: Table S1. Example quotes of participants’ fear of HIV and the impact a HIV diagnosis would have on their lives. [file 12879_2022_7174_MOESM1_ESM.docx]

Additional file 1: Table S1. Example quotes of participants’ fear of HIV and the impact a HIV diagnosis would have on their lives

| Impact of HIV |  |
| --- | --- |
| End of sex life | Yes of course, of course, it may impact my sex life and partner. So if I’m diagnosed, in my opinion, I’m thinking not to have sex at all. I know that it might transmit to someone even if you use a condom, I don’t know sometimes the leaking or not, or whatever. I’m not going to be selfish.  —Participant 4, Thailand, 4 years in Australia |
| Impact on friendships | So yeah, I guess, a lot of people would still be afraid. And like, unwilling to, like, either be friends or go further. Yeah.  —Participant 23, Singapore, 8 months in Australia |
| Impact on relationships | …people just want to protect themselves, so yeah, probably they don’t contact me, or they don’t want to have a relationship with me, yeah.  —Participant 5, Taiwan, 3 years in Australia |
| Would have to isolate from others | I don’t know, it’s just the worst thing that can happen to me… I don’t think people or my friends want to come and stay close to me. They probably try to stay away from me…So [I would] go to live somewhere very far away in countryside or live in a temple.  —Participant 22, Laos, 1 year in Australia |
| Would have to navigate social stigma | … it’s not a death sentence anymore… But still, it’s a social stigma.  —Participant 8, Indonesia, 7 months in Australia |
| Managing HIV in country of origin would be difficult | I’d just stay here and I wouldn’t go back to Indonesia... I don’t want to live with HIV there while the stigma is still heavy, you know.  —Participant 8, Indonesia, 7 months in Australia |
